# A comparative scanning electron microscopy study between the effect of an ultrasonic scaler, reciprocating handpiece, and combined approach on the root surface topography in subgingival debridement

**DOI:** 10.1002/cre2.299

**Published:** 2020-06-22

**Authors:** Leonardo Dassatti, Paolo Francesco Manicone, Selenia Lauricella, Roberta Pastorino, Pierfrancesco Filetici, Fabrizio Nicoletti, Antonio D'Addona

**Affiliations:** ^1^ Department of Head and Neck and Sensory Organs, Division of Oral Surgery and Implantology Fondazione Policlinico Universitario Agostino Gemelli IRCCS, Catholic University of the Sacred Heart Rome Italy; ^2^ Section of Hygiene, Institute of Public Health Catholic University of the Sacred Heart Rome Italy

**Keywords:** reciprocating handpiece, root surface debridement, surface roughness, ultrasonic instruments

## Abstract

**Objective:**

This study aimed to analyze the effectiveness of root‐shape inserts mounted on a reciprocating handpiece during the procedure of root surface debridement (RSD) on extracted teeth. Three different approaches were compared: ultrasonic scaling, employment of root‐shape inserts mounted on a reciprocating handpiece, and a combination of both.

**Materials and Methods:**

A total of 51 extracted teeth were divided into three groups. The first group was instrumented with an ultrasonic scaler, the second group with flexible root‐shape inserts mounted on a reciprocating handpiece (grain size 40, 15, and 4 μm), whereas the final group underwent a combination of both approaches. The time required for the instrumentation was taken. The specimens were subjected to optical and scanning electron microscopy (SEM), and the photographs were evaluated by three examiners who were blinded to the study. The parameters included were: SEM roughness index (SRI) for the roughness calculation, remaining calculus Index (RCI) to evaluate the residual calculus deposits, and loss of tooth substance index (LTSI) to evaluate the loss of tooth substance caused by instrumentation.

**Result:**

The results revealed that the time taken for the instrumentation was on average longer when the root‐shape inserts were employed alone, meanwhile the combined approach did not show significant difference in comparison with the ultrasonic scaling. The lower average RCI was obtained with a combined approach. The use of root‐shape inserts seems to cause a moderate increase in LTSI, especially in a combined approach, whereas it resulted in a better average SRI.

**Conclusion:**

The employment of root‐shape inserts seems to be effective in the RSD for its ability to obtain a smooth and calculus‐free instrumented surface, especially when used in combination with an ultrasonic scaler, and their use can so represent a valid approach to be tested in further in vivo studies.

## 1 INTRODUCTION

Periodontitis is an oral disease that, if left untreated, leads to the destruction of periodontal tissues and can cause tooth loss (Lie & Meyer, 1977). The first step of periodontal treatment is the root surface debridement (RSD), which consists of the removal of the main etiological factors corresponding to plaque and calculus from the tooth surface and obtaining a biologically acceptable root surface while protecting the healthy dental tissues (Arabaci, Cicek, & Canakci, 2007; Chan, Needleman, & Clifford, 2000). Dental plaque or biofilm is composed of microcolonies of bacterial cells, nonrandomly distributed in a shaped matrix or glycocalyx, which subsequently mineralizes to form calculus (Kumar, Swarga Jyoti, Sonowal, & Chawla, 2015). Periodontopathogenic bacteria secrete lipopolysaccharide (LPS), an endotoxin which is absorbed by the most superficial layer of root cementum (Aspriello, Piemontese, Levrini, & Sauro, 2011; Gibbons, 1989; Strachan et al., 2018).

Aleo, De Renzis, and Faber (1975) indicated that human gingival fibroblasts did not adhere in vitro to a root surface contaminated with LPS, and assessed that this was possible only when the cementum was removed by root planing. Many studies tried to determine the presence of endotoxins on the root cementum or even in dentine (Hatfield & Baumhammers, 1971; Nakib, Bissada, Simmelink, & Goldstine, 1982). Saygin, Giannobile, and Somerman (2000) reported that endotoxins were not located within the cementum, concluding that the removal of contaminated cementum was not necessary for successful periodontal treatment and, on the contrary, preservation of the cementum should be preferred to promote a possible new attachment and to save an important source of growth factors (Saygin et al., 2000). Furthermore, it has been demonstrated that cementum plays an important regulatory role in periodontal regeneration (Grzesik & Narayanan, 2002).

The periodic mechanical removal of subgingival microbial biofilm is essential for controlling inflammatory periodontal disease (Arabaci et al., 2007; Graziani, Karapetsa, Alonso, & Herrera, 2017). Subgingival debridement is thus a key therapy in the treatment of periodontitis, either during surgical intervention or in a nonsurgical approach (Aspriello et al., 2011). Most employed devices in periodontal therapy are hand scalers and curettes or ultrasonic scaling instruments (Lie & Meyer, 1977; Singh, Uppoor, & Nayak, 2012). Curettes have some advantages, like the tactile sense of the operator, but manual scaling has some limitations: it is time‐consuming, can cause bleeding, pain and discomfort to patients, the efficacy of the treatment depends on the individual skills of the operator and can lead to excessive removal of dental tissue and formation of a smear layer, which impairs periodontal regeneration (Amid, Kadkhodazadeh, Fekrazad, & Hajizadeh, 2012; Mishra & Prakash, 2013; Tsurumaki et al., 2011).

On the other hand, sonic and ultrasonic scalers have advantages including access to the furcation and deep pockets, less tiring for the operator and less time‐consuming (Kumar et al., 2015).

Several studies have shown the effects of different instruments on root surfaces, assessing that periodontal treatment can be performed less aggressively, avoiding to remove an unnecessary amount of cementum, that can also lead to the exposure of dentinal tubules and cause dental hypersensitivity (Kocher, Fanghänel, Sawaf, & Litz, 2001; Obeid & Bercy, 2005; Obeid, D'Hoore, & Bercy, 2004; Solís Moreno et al., 2012).

Root surface roughness and loss of tooth substance are the possible alterations described in literature after instrumentation, particularly in supportive periodontal therapy (Kumar et al., 2015; Oda, Nitta, Setoguchi, Izumi, & Ishikawa, 2004). The presence of post‐treatment surface roughness would result in greater adhesion of bacterial plaque (Johnson & Brännström, 1974).

For these reasons, the goal of periodontal therapy is to obtain a surface free of bacteria, with less loss of tooth substance as possible and also a smooth and hard tooth surface (Chace, 1974).

The aim of this in vitro study was to assess the efficacy of root‐shape inserts mounted on a reciprocating handpiece in the RSD procedure, comparing the microscopic topography of extracted teeth roots treated with three different approaches: (a) an ultrasonic scaler with periodontal tip, (b) root‐shape inserts mounted on a reciprocating handpiece, and (c) the combination of the two systems.

## 2 MATERIALS AND METHODS

### 2.1 Study population and methodology

#### 2.1.1 Study design

The authors of this research designed and implemented a protocol carried out between April 2018 and February 2019, at the Fondazione Policlinico Universitario Agostino Gemelli IRCCS, Università Cattolica del Sacro Cuore, Rome, with the collaboration between the Periodontal Department of the Dental Clinic with the support of the Dental Institute, SEM Lab.

**FIGURE 1:**
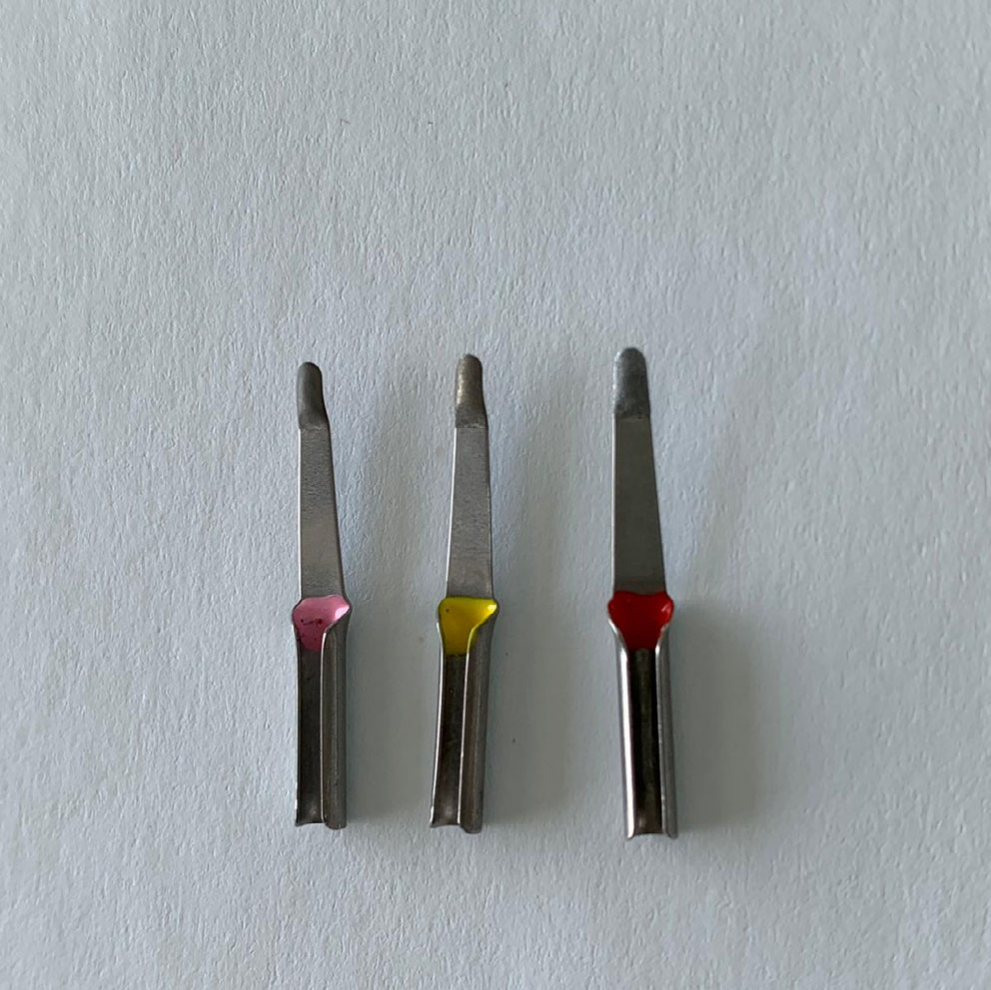
Root‐shape inserts employed in the study (Root Shape Diamond Files, Intensiv SA, Switzerland)

Two different instruments were tested in three different approaches with the creation of three treatment groups:

- Ultrasonic scaler with periodontal tip (HU‐Friedy, Frankfurt, Deutschland).
- Root‐shape inserts used in an oscillating low‐speed handpiece (Root Shape Diamond Files, Intensiv SA, Switzerland; Intensiv Swingle, Intensiv SA, Switzerland). The inserts tested in the present study have three different grain sizes: 40, 15, and 4 μm (Figure 1).
- Ultrasonic scaler with periodontal tip and root‐shape files.

The effects of instrumentation were evaluated according to the following parameters:

- The ability to remove plaque and calculus, with RCI (Zafar, 2016).

**Remaining calculus index**
0: No calculus remaining on the root surface.
1: Small patches of extraneous material, probably consisting of calculus.
2: Definite patches of calculus confined to smaller areas.
3: Considerable amount of remaining calculus, appearing as one or a few voluminous patches or as several smaller patches scattered on the treated surface.

- Loss of tooth substance, enamel and dentine, with loss of tooth substance index (LTSI) (Zafar, 2016).

**Loss of tooth substance index**
0: No delectable loss of tooth substance.
1: Slight loss of tooth substance restricted to localized areas. Most of the cementum is intact.
2: Definite loss of tooth substance on most of the treated surface, but without deep instrumental marks in the dentine. Cementum may be absent in some areas.
3: Considerable loss of tooth substance with deep instrumental marks in the dentin. Most of the cementum is removed.

- The residual root surface roughness, with scanning electron microscopy (SEM) roughness index (SRI) (Johnson & Brännström, 1974).

**Scanning electron microscopy roughness index**
0: Smooth and even surfaces or slightly roughened, but without signs of instrumental marks.
1: Slightly roughened areas with some corrugated regions but no obvious instrumental marks.
2: Definitely corrugated areas and some instrumental marks, but also relatively even areas.
3: Definitely corrugated surface with instrumental scratches over most of the areas

#### 2.1.2 Experimental procedure

The in vitro study was conducted on 51 human teeth extracted either for periodontal or orthodontic reasons. Extractions were performed without the use of levers to avoid the presence of cracks on the surfaces and, after the surgical procedure, teeth were immediately rinsed with saline solution and were then stored in a saturated water solution, about 10% formaldehyde by volume. Specimens were divided into three groups of 17 teeth, each group corresponding to the instrumentation approach to be tested. A sample size of 17 for each group is necessary to achieve a power of 0.80 (*α* = .05) with a delta for the variable SRI of 0.44. Teeth were allocated in the test groups to obtain homogenous groups in terms of the amount of proximal calculus and similar root morphology as assessed by the naked eye.

A test area was marked on the proximal surface of the tooth by two grooves in facio‐lingual direction with a distance of 5 mm from each other.

Root‐shape files were used following the recommendations for use and setting the micromotor at 40,000 rpm/min max that corresponds, with the oscillating low‐speed handpiece, to approximately 20,000 oscillations per minute (2:1). An adequate water spray cooling was provided, with minimum flow of 50 mL/min. The root‐shape inserts have three different grain sizes: 40, 15, and 4 μm, which have been used in succession as suggested by the company. A load of around 50 g was applied so as not to bend the instrument during its use.

The root treatment, operated with a ×4.5 magnification system, stopped as soon as the test surface seemed smooth and clean by visual and tactile judgment with a periodontal probe.

The approximate time required to clean each surface was noted.

Upon completion of the test procedures, the teeth were rinsed in running tap water and dehydrated through ascending grades of ethyl alcohol (70, 95, and 100% × 2) followed by air drying for 48 hr. Only one trained operator performed all the procedures to eliminate interoperator variability and minimize variables such as stroke length, force and pressure applied during instrumentation.

Teeth were then fixed to SEM stubs with conductive double‐sided tape and with silver paint, the specimens were then sputter‐coated with 30–40 nm of gold and palladium during continuous tilting and rotation of the specimen.

#### 2.1.3 Optical microscopy

After the dehydration procedure, the teeth were analyzed with a stereomicroscope *Nikon SMZ 745 T*, and photos were taken with magnification at ×6, ×7, and ×10, and three photos with ×20, in order to have another verification parameter for the calculation of RCI and LTSI.

#### 2.1.4 Scanning electron microscopy

Five standardized micrographs were taken with SEM *ZEISS ESEM 25*, one low magnification at ×55 at the central part of the test area to inspect the entire treated area and avoiding so the possibility of being biased by the selection, and four micrographs with magnification at 100×.

The optical microscopy and SEM photographs were interpreted by three examiners who were blinded to the study and the data obtained were then subjected for statistical analysis.

#### 2.1.5 Statistical analysis

Interrater reliability was assessed using Fleiss' Kappa. Statistical differences in RCI, SRI, and LTSI among the three approaches were determined by Kruskal Wallis (a *p* value < .05 was considered to indicate a statistically significant difference), while post hoc tests were performed using Bonferroni correction to compare results of two approaches at a time. The statistical difference for the time taken for instrumentation was determined by one‐way analysis of variance (ANOVA, significant *p* value < .05).

## 3 RESULTS

**FIGURE 2:**
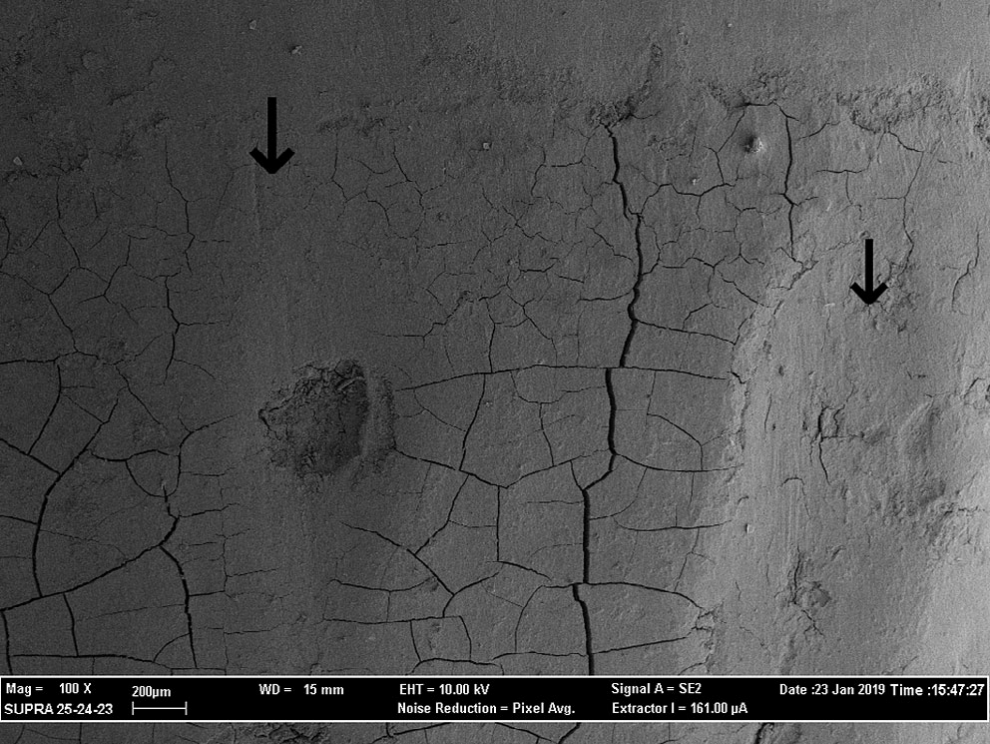
SEM (magnification ×100) showing the morphology of the root surfaces treated by root‐shape inserts mounted on a reciprocating handpiece. Microcracks should appear on the dehydrated cementum, their absence shows some loss of tooth substance (arrows). SEM, scanning electron microscopy

**FIGURE 3:**
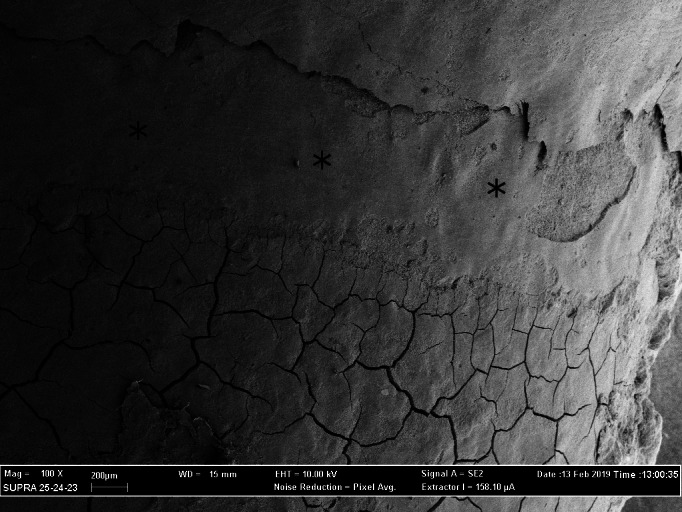
SEM (magnification ×100) showing the morphology of the root surfaces treated by an ultrasonic scaler. Image shows instrumental marks caused by the scaler (asterisk). SEM, scanning electron microscopy

**FIGURE 4:**
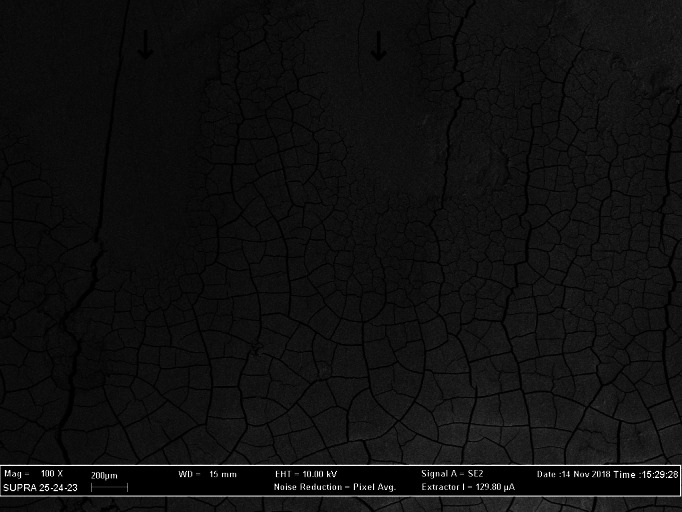
SEM (magnification ×100) showing the morphology of the root surfaces treated by a combined approach, resulting in a very low surface roughness. Some loss of tooth substance occurred (arrows). SEM, scanning electron microscopy

The cracks consistently present in the surface layer of the cementum represented artifacts produced by the dehydration during specimen processing (Figures 2, 3, and 4). Their presence was of considerable value both concerning the distinction between cementum and calculus and in the assessment of the amount of lost tooth substance, since neither the calculus nor dentine displayed such cracks.

Average, median, and *SD* values for all the parameters evaluated in the study are reported in Table 1.

**TABLE 1:** Synthesis of the results

	Root‐shape files	Ultrasonic scaling	Combined
**RCI**			
Average	0.78	1.22	0.37
Median	0.33	0.67	0.00
*SD*	0.90	1.05	0.81
**SRI**			
Average	0.86	1.80	0.55
Median	1.00	2.00	0.33
*SD*	0.54	0.82	0.36
**LTSI**			
Average	1.88	1.39	1.84
Median	2.00	1.00	2.00
*SD*	0.89	0.77	0.75
**TIME**			
Average	87.55	44.36	49.09
Median	89.10	45.00	51.40
*SD*	8.86	7.16	8.88

*Note: SD* values are high for RCI, SRI, and LTSI for their intrinsic quality of being short‐range score scales.

Abbreviations: LTSI, loss of tooth substance index; RCI, remaining calculus Index; SRI, SEM roughness index; TIME, time needed for instrumentation.

### 3.1 Instrumentation time

The time required to clean the test surfaces differed markedly in the three different groups (*p* < .0001). The instrumentation with root‐shape files alone had the longest average scaling time (87.55 s), whereas the ultrasonic system and the combined approach required shorter and similar times (44.36 and 49.09 s, respectively; *p* = .345) (Table 1).

### 3.2 Removal of calculus

Removal of calculus (RCI) scores revealed that all the instrumentation procedures were effective in removing root surface calculus. Large remaining deposits were rarely seen. Thin layers of calculus were sometimes seen in local areas or more scattered over a larger part of the test surface. All the analyzed approaches showed low average and median values, but a borderline global significant difference was found through Kruskal Wallis analysis (*p* = .06) (Table 1).

The combined approach tended to remove calculus more completely than the ultrasonic alone (average RCI 0.37 and 1.22, respectively) with a statistically significant difference calculated through the post hoc Bonferroni analysis. No significant differences emerged between the combined approach and instrumentation with root‐shape files (average RCI 0.78) (Table 2).

**TABLE 2:** Representation of the statistical analysis of the averages RCI (blue), SRI (yellow), and LTSI (green) performed through Bonferroni post hoc test to find significant differences (numbers in bold) between two approaches at a time

	Ultrasonic scaling	Root‐shape files	Combined		RCI
Ultrasonic scaling		**0.0032**	**<0.0001**		SRI
Root‐shape files	0.19		0.35		LTSI
Combined	**0.03**	0.63			
		0.14	**0.09**	Ultrasonic scaling	
			0.99	Root‐shape files	

Abbreviations: LTSI, Loss of tooth substance index; RCI, Remaining calculus Index; SRI, SEM roughness index.

### 3.3 Loss of tooth substance index

LTSI scores revealed that the instrumentation with root‐shape files and the combined approach presented an average greater loss of tooth substance than the ultrasonic alone (average LTSI 1.88, 1.84, and 1.39 respectively) (Table 1); nevertheless, a significant difference could only be found for what concerns the comparison between the ultrasonic scaling and the combined approach (Table 2). Global *p* value was notsignificant (*p* = .12).

### 3.4 Surface roughness index

SRI scores revealed a significant difference between the three methods (global *p* value < .0001).

The combined approach presented the average smoothest surfaces (average SRI 0.55). The ultrasonic scaling alone resulted in the higher SRI scores (average SRI 1.80) (Table 1), with significant differences with both of the other approaches (Table 2).

### 3.5 Interrater reliability

Interrater agreement scores are represented in Table 3. Concordance between the ratings is significant, thus it can be assessed that the hypothesis that they are making their determinations randomly is rejected.

**TABLE 3:** Interrater agreement percentages

	RCI (%)	SRI (%)	LTSI (%)
Examiners 1–2	74.51	62.75	56.86
Examiners 1–3	72.55	45.10	41.18
Examiners 2–3	58.82	54.90	56.86

Abbreviations: LTSI, Loss of tooth substance index; RCI, Remaining calculus Index; SRI, SEM roughness index.

## 4 DISCUSSION

This in vitro study evaluated the efficacy of root‐shape inserts considering several parameters that characterize RSD, thus focalizing on different aspects of the treatment.

For what concerns the ability to remove calculus, RCI scores in this study showed a good behavior of root‐shape inserts, resulting in a mildly better average if compared to the ultrasonic scaler, while the most effective approach was the combined use of both.

The employment of root‐shape inserts seemed to increase the loss of tooth substance (measured through LTSI), that was anyway moderately significant only when they were additionally used after an ultrasonic instrumentation.

A significant improvement was observed, instead, in the smoothness of the instrumented surface when the root‐shape files were employed alone or, with a strong statistical evidence, in a combined approach.

There are only few and dated in vitro studies about the role of reciprocating handpieces in the RSD and none of them employed the very same inserts of the present work. Jotikasthira, Lie, and Leknes (1992) and Lee, Heasman, and Kelly (1996) tested the same reciprocating system evaluating parameters close to the ones used for the present work, but combining SRI and LTSI in a unique parameter called Roughness and Loss of Tooth Substance Index (RLTSI). These works found good results for what concerns both RCI and RLTSI and did not detect differences between the reciprocating system and ultrasonic scalers. Their results, however, are not fully comparable to the ones obtained in this study for the different inserts employed on the handpieces in their works, which were cutting‐edges instruments instead of diamond files (Jotikasthira, Lie, & Leknes, 1992; Lee, Heasman, & Kelly, 1996). Obeid and Bercy (2005) studied another reciprocating system which works in two phases with a scaler‐like insert at first and a diamond file to polish the surface in the end. In this study, the authors only evaluated loss of tooth substance caused by instrumentation, finding notsignificant differences between this system and ultrasonic scalers. The same group also tested this reciprocating system in a clinical trial, assessing the same efficacy in RSD than more conventional approaches such as instrumentation with ultrasonic scalers or curettes (Obeid et al., 2004).

An important finding of this study is the low roughness detected on surfaces treated with root‐shape inserts, as it was demonstrated that rough surfaces are related to higher biofilm accumulation. Thus, obtaining a smooth surface is considered mandatory in RSD to avoid a fast plaque formation in the post‐treatment period (Folwaczny, George, Thiele, Mehl, & Hickel, 2002; Kocher, Rosin, Langenbeck, & Bernhardt, 2001).

Ultrasonic scalers and hand instruments were found to have the same efficacy in RSD, so it cannot be assessed the superiority of one in respect to the other, but ultrasonic scalers seem to leave a rougher treated site than curettes, therefore, other in vitro studies comparing root‐shape files and hand instruments, especially for what concerns surface roughness parameters, could offer a wider overview on this topic (Meyer & Lie, 1977; Obeid et al., 2004).

The SRI (or the above mentioned RLSTI) is a widely employed parameter to analyze the residual roughness of an instrumented surface, but other methods, such as a profilometer evaluation, could help in further studies to have a nonsubjective analysis of the specimens (Lie & Leknes, 1985; Vastardis, Yukna, Rice, & Mercante, 2005).

Despite the employment of root‐shape files alone can be considered since its high efficacy in the removal of calculus demonstrated by the low RCI scores in this study, the long instrumentation time required to reach a complete cleaning might lead to a better use of these instruments in a combined approach with other less time‐consuming devices such as ultrasonic scalers. There might also be the possibility that root‐shape files instrumentation resulted in such low scores concerning RCI due to the intrinsic instrument's tendency to “polish” calculus rather than remove it, therefore making them indistinguishable from the root surface in a SEM observation. On the other side, when visual cleanliness and tactile smoothness are used as the endpoints RSD, consideration should be given to the tendency toward over instrumentation as it can often be difficult to distinguish between burnished calculus and roughened, but clean, cementum.

Such problems may also unnecessarily lengthen the time taken for root debridement with an instrument.

The parameters evaluated in this study have been already used in many articles and for this reason they are considered appropriate and suitable even if using scales with a low range can be considered as study limitation, leading to possible high discordance between the examiners (Kishida, Sato, & Ito, 2004; Marda, Prakash, Devaraj, & Vastardis, 2012). However, by comparing the data, the interrater agreement resulted in a significant concordance.

Due to the obvious limits of an in vitro study, it is necessary to test the clinical effects of the root‐shape inserts with in vivo clinical trials.

In vivo studies could also provide further information about the combined approach that has been proposed in this study.

## 5 CONCLUSION

Instrumentation with root‐shape inserts showed efficacy in the removal of calculus and in the smoothening of the surface in an in vitro RSD procedure, resulting in similar or better results when compared to ultrasonic scaling. A combined approach involving both the systems seemed convenient to reduce the long instrumentation time required by the root‐shape inserts when employed alone, even if this approach led to a mildly higher loss of tooth substance.

Further in vivo studies are required to validate the efficacy of the inserts in clinical practice.

### CLINICAL RELEVANCE

#### Scientific rationale for study

There is very poor evidence about inserts mounted on reciprocating handpieces in periodontal treatment.

#### Principal findings

Proof of efficacy of these inserts, especially when combined with ultrasonic scaling.

#### Practical implications

Employment of root‐shape inserts mounted on a reciprocating handpiece after the ultrasonic scaling of the root can lead to a better calculus removal and a lower surface roughness. A randomized clinical trial is required to confirm these in vitro findings.

## AUTHOR CONTRIBUTIONS

Leonardo Dassatti and Paolo Francesco Manicone conceived the ideas. Selenia Lauricella and Fabrizio Nicoletti collected the data. Roberta Pastorino analyzed the data. Selenia Lauricella and Pierfrancesco Filetici led the writing. Antonio D'Addona critically reviewed the writing.
